# Social Media Perceptions of Surgical Cancer Care in the Era of COVID-19: A Global Cross-Sectional Study

**DOI:** 10.1200/GO.20.00326

**Published:** 2020-08-14

**Authors:** Evan J. Keil, Sergio M. Navarro, Hashim Shaikh, Lilian E. Yao, Todd M. Tuttle

**Affiliations:** ^1^ Department of Surgery, University of Minnesota, Minneapolis, MN; ^2^ Department of Surgery, University of California, San Francisco, San Francisco, CA

## Abstract

**PURPOSE:**

The rapid dissemination of information through social media renders a profound lens to evaluate perceptions of emerging topics, especially in the context of a global pandemic. The primary objective of this cross-sectional study was to elucidate trends on social media in the setting of surgical cancer care affected by the COVID-19 pandemic across the globe.

**METHODS:**

A public search of Twitter from April 1 to 30, 2020, was conducted, which yielded 996 posts related to COVID-19 and cancer. Two authors (E.J.K. and H.S.) individually reviewed all posts and recorded the post category, engagement, author category, and geographic location. Data were then analyzed through descriptive analyses. Only English-language posts were included, and any noncancer- or non-COVID–related posts were excluded from the analysis.

**RESULTS:**

A total of 734 unique authors from 26 different countries wrote 996 relevant posts that averaged 12.0 likes, 4.7 retweets, and 0.5 hashtags per post. Only 2.3% (23 of 996) of posts included a video. Authors of the included tweets most frequently were friends and families of patients (183; 18.4%), academic institutions or organizations (182; 18.3%), and physicians (138; 13.9%). Topics of importance were cancellations of surgeries (299; 40.1%), COVID-19 education (211; 121.2%), and research studies (93; 9.3%). The United Kingdom and the United States made up 81.5% of the cohort, followed by Canada (6.6%) and India (2.4%). Of posts where a specific type of surgery was identified (196), the most common type mentioned was breast cancer (50; 25.5%), followed by lung cancer (37; 18.9%) and urologic cancer (22; 11.2%).

**CONCLUSION:**

This analysis provides insight into the resulting impacts of COVID-19 on the global discussion of surgical cancer care.

## INTRODUCTION

The use of social media as a means to evaluate individuals’ perceptions of varying topics has been widely used within the medical community and is an easily accessible tool for rapid dissemination of information. On specific platforms, namely, Twitter and Instagram, there exists a wide range of individuals from across the globe who actively discuss topics as they are developing. Twitter has been shown, in certain spheres, to have a more academic lens through which emerging topics are discussed and posted.^1^ Other platforms, such as Instagram and Facebook, tend to be centered around personalized stories and are community based.^2^ The use of social media has been rising globally, and thus, social media may allow for a holistic view of how topics affect the world, although conversations continue to be dominated by higher-income countries.^3^ Moreover, the ability to identify trends in a rapid fashion and how they change over time further corroborates the use of social media analyses within the medical community. While data have been published in survey form on the basis of an evaluation of the impact to cancer surgery from the perspective of surgical oncologists, there is a need to evaluate the impact on and perspectives of the wider surgical cancer community.^4,5^

**CONTEXT**

**Key Objective**
What is the impact of COVID-19 on the surgical oncologic social media community?
**Knowledge Generated**
Main communities represented were breast cancer, lung cancer, and urologic cancer, with equal input from patients, families, academic institutions, and physicians. There was an overwhelming perception that cancer surgery is essential and a focus on the cancellations, rescheduling, and need for education with regard to best practices within cancer care during the global pandemic.
**Relevance**
Patients and families are active on social media, and analysis of trends within these communities may allow physicians and academic institutions to respond to the rapidly evolving COVID-19 pandemic and further improve patient care.

The coronavirus disease 2019 (COVID-19) has drastically altered the face of society, including medicine, across the globe from its initial outbreak in Wuhan, China, in December 2019 to its eventual evolution into a global pandemic. Surgeries have been canceled, treatment plans have been altered, and delays are present across nearly all specialties.^6-10^

The ability to provide surgical cancer care during this pandemic has affected many, as reflected through social media. The primary focus of this study was to perform a cross-sectional, observational study to elucidate the impact of COVID-19 on surgical cancer care across the globe over a specific time period.

## METHODS

A search of Twitter was performed on May 15, 2020, for public posts within a 1-month time period: April 1-30, 2020. Posts were identified by a search of the public domain within that time period and by two relevant hashtags: #CovidCancerSurgery and #CoronaCancerSurgery. Given the expansive topic of COVID-19 and the rapidly evolving literature and public discourse, a quantitative approach was taken to identifying the hashtags using the Symplur Healthcare Hashtag Project.^11^ Other hashtags, such as #cancersurgery, #covidcancer, #covid, #oncology, #covidsurg, and #covid19ncancer, among others, were considered; however, the two that were included had the most specific and highest yield with regard to number of posts and relevance to the topic at hand. Multiple other social media platforms were considered for evaluation; however, Twitter provided the most accessible and readily searchable platform for an analysis of this nature.

The query of the public domain revealed 996 posts, all of which met the inclusion criteria. Only posts related to human participants were included in the analysis. Only English-language posts were included to avoid misinterpretation and because of a lack of funding for qualified interpreters. Posts were analyzed for inclusion and data collection by two separate authors (H.S. and L.E.Y.). No posts were excluded from the analysis because of inter-rater variability. A review of the original media was conducted with the first author to achieve agreement.

Data were collected on each of the 996 included posts for the presence of photos or videos; engagement; author type; post content; type of cancer, if mentioned; and geographic location. All categories, with the exception of post content, were rated with a binary scoring system that did not allow for multiple categories to be chosen for content type, author type, and location. In terms of author type, eight representative categories were chosen: patient, patient’s friends and family, business, news organization, nurse, physician, academic organization or institution, and other. For post content, posts were allocated to one or more of 15 representative categories. Categories were chosen through review of previous social media analysis of medical topics and discussion among the authors. For example, if a post discussed both the cancellation of surgery and the receipt of treatment alone, it was recorded as such. Engagement was recorded by the number of likes, retweets, and hashtags present with each post as of the review date (May 17, 2020). Location data were collected through the evaluation of tagged geographic data as well as through a thorough review of the author biographies. Data were analyzed with descriptive analysis and categorical analyses.

## RESULTS

A total of 996 posts were identified on Twitter during April 2020 on the basis of the aforementioned search criteria. There were 734 unique authors with a mean of 1.36 posts per author. Of these included posts, 23 included a video (2.3%), while all other posts included only a photo or text. In terms of visibility and engagement, posts had an average of 12 likes per tweet and 4.7 retweets. An average of 0.5 hashtags was present in each of the tweets. Of the representative categories of authors, tweets were most frequently posted by friends and family of patients (183; 18.4%), academic institutions or organizations (182; 18.3%), physicians (138; 13.9%), news organizations (122; 12.2%), and patients (114; 11.4%). Categorical data are listed in Table 1.

**TABLE 1:**
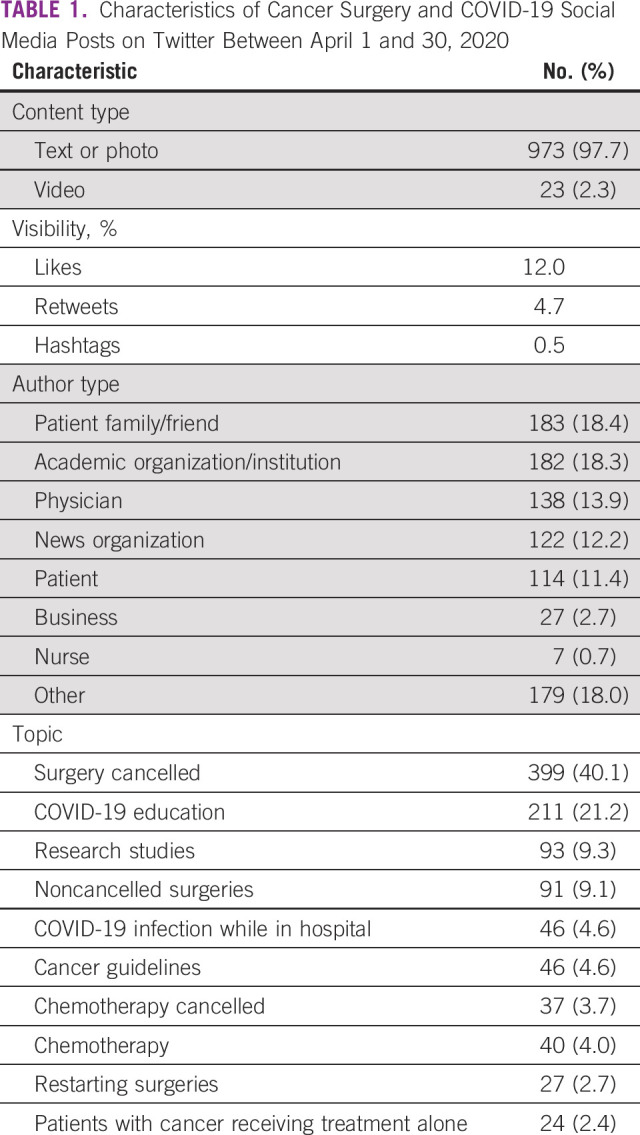
Characteristics of Cancer Surgery and COVID-19 Social Media Posts on Twitter Between April 1 and 30, 2020

Topics of discussion were categorized into 15 representative groups, including surgery cancellations, COVID-19 education, research studies, noncancelled cancer surgery, COVID-19 infection while in the hospital, cancer guidelines, chemotherapy guidelines, chemotherapy, and restarting surgeries, among other categories. Tweets most frequently focused on cancellations of surgeries (399; 40.1%), followed by education on COVID-19–related items (211; 21.2%), research studies (93; 9.3%), noncancelled cancer surgeries (91; 9.1%), and COVID-19 infection in the hospital (46; 4.6%; Table 1).

Research studies (n = 126) were shared most frequently by academic institutions and organizations (82; 65%), followed by physicians (12; 9.5%). Educational information on COVID-19 (n = 211) was shared most frequently by academic institutions and organizations (133; 63.0%), followed also by physicians (22; 10.4%). Patients and their friends and families posted a total of 11 times (1.10%) on research studies and COVID-19 educational content.

For a global review, geographic information with regard to the post’s country of origin was only available in 579 (58.1%) of the 996 posts (Table 2). Twenty-six different countries were represented within the cohort. The United Kingdom and the United States made up 81.5% of the cohort (472 of 579), followed by Canada (38; 6.6%) and India (14; 2.4%). Within the United States, the specific state was determined in 200 of the 229 posts, with New York (23), California (21), and Florida (18) making up the highest number of posts (Table 3).

**TABLE 2:**
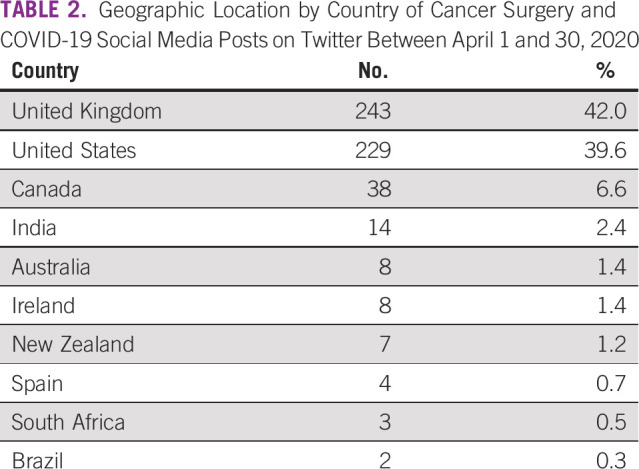
Geographic Location by Country of Cancer Surgery and COVID-19 Social Media Posts on Twitter Between April 1 and 30, 2020

**TABLE 3:**
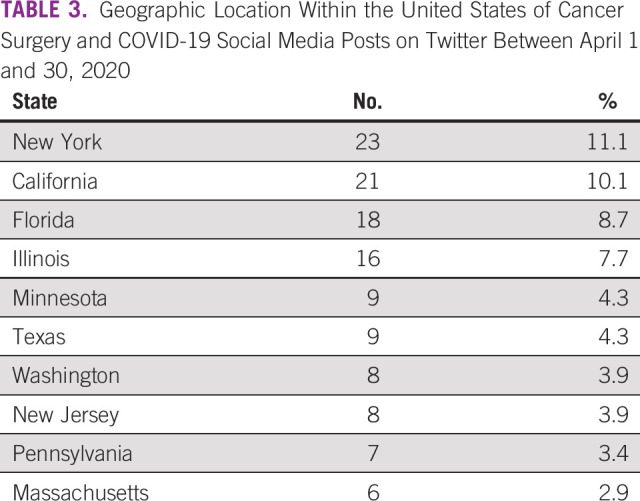
Geographic Location Within the United States of Cancer Surgery and COVID-19 Social Media Posts on Twitter Between April 1 and 30, 2020

**FIG 1:**
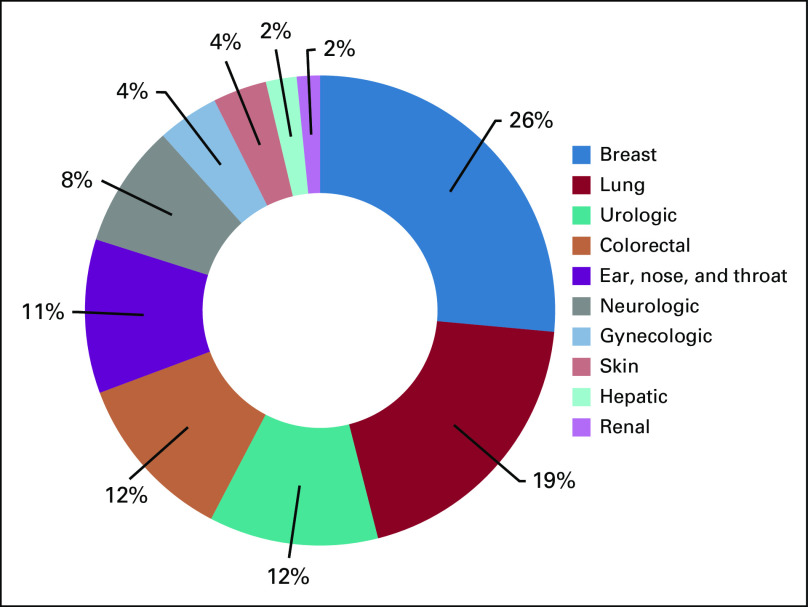
Subspecialty mentions in Tweets related to cancer surgery and COVID-19 social media posts on Twitter between April 1 and 30, 2020.

Of the 996 included posts, 196 (19.7%) mentioned a specific type of cancer. Of these, the most common type mentioned was breast cancer (50; 25.5%), followed by lung cancer (37; 18.9), urologic cancer (22; 11.2%), colorectal cancer (22; 11.2%), and otorhinolaryngologic cancer (20; 10.2%). The leading subspecialty mentions are summarized in Figure 1.

## DISCUSSION

The primary objective of this observational, cross-sectional study was to illuminate the impact of the COVID-19 pandemic on cancer care and surgery across the globe through an analysis of social media. Perspectives from academic institutions, patients, friends and family, businesses, and physicians highlight such topics as cancellation of surgery; COVID-19 education; research studies; and, less frequently, the resumption of surgery and cancer guidelines.

The cancellation of cancer surgeries and appointments is of the utmost importance and accounted for more than one third (34.7%) of the content posted on Twitter about cancer care, surgery, and COVID-19 in the month of April 2020. Patients within the cohort focused on the cancellation of their surgeries 46.5% (40 of 86) of the time, and physicians posted about cancellation 33.4% of the time. Posts often had a fearful or angry tone about the cancellation of surgeries. One individual from the United States was diagnosed with invasive breast cancer at the beginning of March and had her surgery postponed indefinitely because of COVID-19 concerns. Another individual from South Africa posted frustration with the cancellation of melanoma resection and chemotherapy course, stating “If I don’t die from a virus, I will certainly die from untreated cancer.” Others focused on what is truly nonessential and how difficult this definition can be. In our analysis of the Twitter posts, the pandemic has left both physicians and patients with numerous cancellations and delays, an unacceptable position for many.

In contrast, 91 tweets discussed the continuation of cancer surgeries despite the COVID-19 lockdowns and delays others had been experiencing. Many of these posts highlighted negative COVID-19 test results, which allowed them or their family members to receive their much-needed surgery. Given the contrast between these 91 tweets and a majority of others included in our study, there exists a dichotomy created within the care of patients with cancer as a result of the COVID-19 pandemic. For some, surgeries have been delayed for multiple months, and for others, their surgeries are still on track.

The safe return to clinics and operating rooms were mentioned only 24 times throughout the tweets analyzed (2.4%); however, the way in which the return to clinics is handled will likely influence not only these patient’s health outcomes but also their overall perceptions of the health care community.^12^ Twenty of the 24 posts that spoke about the return to surgeries occurred after April 14, which indicates an increase in its popularity among physicians and patients alike. Physicians who discussed the return to surgery highlighted the need for personal protective equipment and updated guidelines as well as simply the need for cancer surgery to continue because it is overwhelmingly considered as essential from both the provider and the patient perspective.^13,14^ The return to clinics must be addressed in a safe, efficient, and quick manner because cancellations and the return to surgery were emphasized by all cohorts in our study.^15^

Research articles (93; 9.3%) and educational information (211; 21.2%) on the COVID-19 pandemic made up approximately one third of the overall discussion surrounding cancer care, surgeries, and coronavirus. Individuals from all author types shared content held within these categories; however, academic institutions and organizations shared this information most frequently, followed by physicians, in terms of the total number of posts shared within the cohort. Educational information was often shared through descriptive graphics containing country-specific information or guidelines. Twitter served as a platform for these organizations and physicians to share information and discuss the most frequent research findings and their implications on patients and populations as a whole. However, patients and their friends and family overwhelmingly did not participate in discussions of this nature, accounting for only 11 research or educational posts. Patients and their friends and families made up 29.8% of authors, yet there was little overlap in content between them and what physicians and organizations were sharing. Organizations and physicians ought to address directly the concerns of patients because Twitter is an accessible platform that allows for direct interaction and the possibility for strengthening relationships with those outside the medical community.

To our knowledge, patient perceptions on cancer surgical care during the era of COVID-19 have not been studied. There exists research on surgical oncologists and how COVID-19 has affected their practices and their ability to treat disease^16^; however, no prior study combines these two important perspectives. While social media allows an immediate view into concerns and desires of patients, providers, and organizations, additional research may be able to more directly address this issue as highlighted here.^17^

Another topic mentioned in 51 (5.6%) of the 996 included tweets was the delay of cancer diagnoses. An important difference exists between these tweets and those of patients who had their surgeries canceled or delayed. In those in whom surgeries were delayed or canceled, presumably a treatment plan is in place. Although delayed or pushed out for multiple months, in many cases, the diagnosis is known and the risks are discussed when canceling surgeries. In addition, many patients with cancer can be offered effective alternatives to immediate surgery; for example, many patients with estrogen receptor–positive breast cancer were given preoperative endocrine therapy during the pandemic as a bridge to surgery in the future. However, patients with undiagnosed cancer who delay presentation because of COVID-19 lockdowns, hospital policies, or a general fear of contracting the virus are at risk for delayed diagnoses and treatments and worse cancer outcomes. This situation has been studied for patients with acute coronary syndrome and stroke^18^; however, to our knowledge, no study has been published with regard to cancer diagnoses. One father advocated for his son to receive a COVID-19 test despite being asymptomatic so that he might be screened for cancer after finding a lump; another woman in the United Kingdom feared the ramifications of people worried about going to their doctor in terms of cancer diagnoses. While cancellations must be addressed, so too must patient fears of contracting the virus from normal checkups and screenings.

The limitations of this study include variation in the use of Twitter in different countries and social media preferences in different populations.^19^ Even given the increase in social media usage in low- and middle-income countries over the past few years, conversations of these types are often dominated by high-income countries, thus limiting the discussion and perspectives included in the analysis.^20^ The decision to use only English-language posts with the search criteria likely influenced the high percentage of posts coming from the United States and United Kingdom, which further biases the analysis. Social media data analyses inherently have limitations as well, with the possibility of oversimplification of complex topics through the use of built-in analytic tools. Using a social media analysis to elucidate trends may not represent the broader opinions of groups of patients, their families, and physicians as well as organizations as a whole.

While our study is cross-sectional and many countries are represented, the data set is limited, and our sample size is small and time frame short compared with the worldwide use of social media; thus, our findings may be limited and not generalizable. Given our short period of study and the rapidly changing environment and discussion around COVID-19, the peak of discussion may not have been captured in different locations, thus limiting our scope. In addition, the use of two hashtags further limits this data set and decreases the ability for trends to be generalized to all cancer surgical care in the era of COVID-19. Finally, our results may not be representative of all age-groups because Twitter use tends to be among younger cohorts.^21^

In conclusion, the COVID-19 pandemic has fundamentally altered the administration of cancer care across the globe. Analysis of a focused subset of social media allows for a detailed view of individuals’ perceptions in the cancer community with regard to emerging issues presented by COVID-19. In this cross-sectional analysis of a single social media platform during the month of April 2020, trends within the discussion of cancer care during the pandemic were elucidated. The cancellation of surgeries was of particular concern for patients, although all categories of authors emphasized this as well. Physicians began discussing return to surgery as well as the need for cancer surgery to continue to occur. Other topics such as education surrounding COVID-19, noncancelled cancer surgeries, and having COVID-19 while recovering in the hospital were also highlighted among authors. These findings provide an indicative sampling of fundamental perceptions of COVID-19 within cancer care and surgeries.
